# Efficacy of Preoperative Pericapsular Nerve Group Block in Patients with Hip Fracture and its Effect on the Success of Spinal Anaesthesia: A Retrospective Study

**DOI:** 10.4274/TJAR.2024.241636

**Published:** 2024-12-16

**Authors:** Burcu Kaplan, Eyyüp Sabri Özden, Mustafa Soner Özcan, Filiz Alkaya Solmaz, Pakize Kırdemir

**Affiliations:** 1 Süleyman Demirel University Faculty of Medicine Department of Anaesthesiology and Reanimation, Isparta, Turkey

**Keywords:** Hip fracture, opioid use, pericapsular nerve group block, spinal anaesthesia

## Abstract

**Objective:**

We intended to research the efficacy of pericapsular nerve group (PENG) block performed with preoperative ultrasonography (USG) in patients who underwent hip fracture repair under spinal anaesthesia and whether it affects the success of spinal anaesthesia.

**Methods:**

The files of 100 patients were analysed, and 60 patients were enrolled in the study. The patients were assigned into two groups: Group P (n= 30) consisted of patients who underwent USG-guided PENG block before the start of surgery and the control group (Group C; n= 30) consisted of patients in whom tramadol infusion was initiated. All patients received 10 mg IV bolus tramadol as rescue analgesia when numeric rating scale (NRS)>3. From the files of the patients, before PENG block application, after PENG block application, during positioning before spinal anaesthesia, the NRS values at the time of the patient’s discharge from the operating room and at 2^nd^, 4^th^, 12^th^ and 24^th^ hour NRS values, spinal anaesthesia duration and number of attempts, and perioperative total tramadol consumption were obtained.

**Results:**

In group P, NRS values were found to be significantly lower after PENG block application, during positioning before spinal anaesthesia, and at the postoperative discharge, 2^nd^, 4^th^, 12^th^ and 24^th^ hours. In addition, group P had a lower duration of spinal anaesthesia, a lower number of spinal anaesthesia attempts and a lower total perioperative tramadol consumption.

**Conclusion:**

The results demonstrated that preoperative PENG block facilitated positioning for spinal anaesthesia, shortened the application time, increased the first-attempt success rate, decreased pain scores, and reduced the need for postoperative opioids.

**Main Points**

• The advantage of the pericapsular nerve group (PENG) block is that it can be applied in the supine position for a patient with pain that worsens with movement, and three nerve blocks can be performed with a single needle insertion.

• PENG block performed on hip fracture patients in the preoperative care unit can provide effective analgesia in patients during preoperative transfer and spinal anaesthesia positioning.

• We found that it provided lower numeric rating scale values in the postoperative period and reduced opioid use and associated side effects.

• When the number of spinal anaesthesia attempts was compared among the groups, the success rate of the first attempt was statistically significantly higher in Group P with 64%.

## Introduction

The aim of surgical treatment in hip fracture patients is to provide long-term mobility and the best possible function while aiming for low disability and mortality rates.^1^ However, the recommended type of anaesthesia is still open to debate.^2^ Among the regional anaesthesia techniques, unilateral hipobaric spinal anaesthesia in lateral decubitus position is a popular choice because it can be applied in the surgical position and causes fewer hemodynamic changes.^3^

It is essential to note that uncontrolled pain, which has the potential to have both physiological and psychological negative effects, can make it challenging to provide a suitable position for spinal anaesthesia and may affect the procedure’s success.^4, 5^ Effective pain control with regional analgesia can lead to faster recovery, shorter hospital stays, and cost-related benefits. Additionally, regional blocks applied under ultrasonography (USG) guidance have fewer side effects.^6, 7^

The pericapsular nerve group (PENG) block was first developed for postoperative analgesia in total hip arthroplasty in 2018.^8^ One of the key benefits of the USG-guided PENG block is that it can be applied in the supine position for a patient with pain that increases with movement. This makes it a particularly suitable option for those who experience discomfort when moving around. Additionally, it is possible to perform a block for the articular branches of the femoral, obturator and accessory obturator nerves with a single needle entry, which is a useful advantage.^9^

The aim of our study was to assess the efficacy of PENG block in patients undergoing hip fracture surgery with spinal anaesthesia. Our primary objective was to demonstrate that the PENG block improves the success of spinal anaesthesia in the first attempt by reducing pain during spinal anaesthetic positioning and shortening the administration time. Our secondary objective was to demonstrate that PENG block reduces postoperative pain, opioid use, and side effects.

## Methods

**Figure 1:**
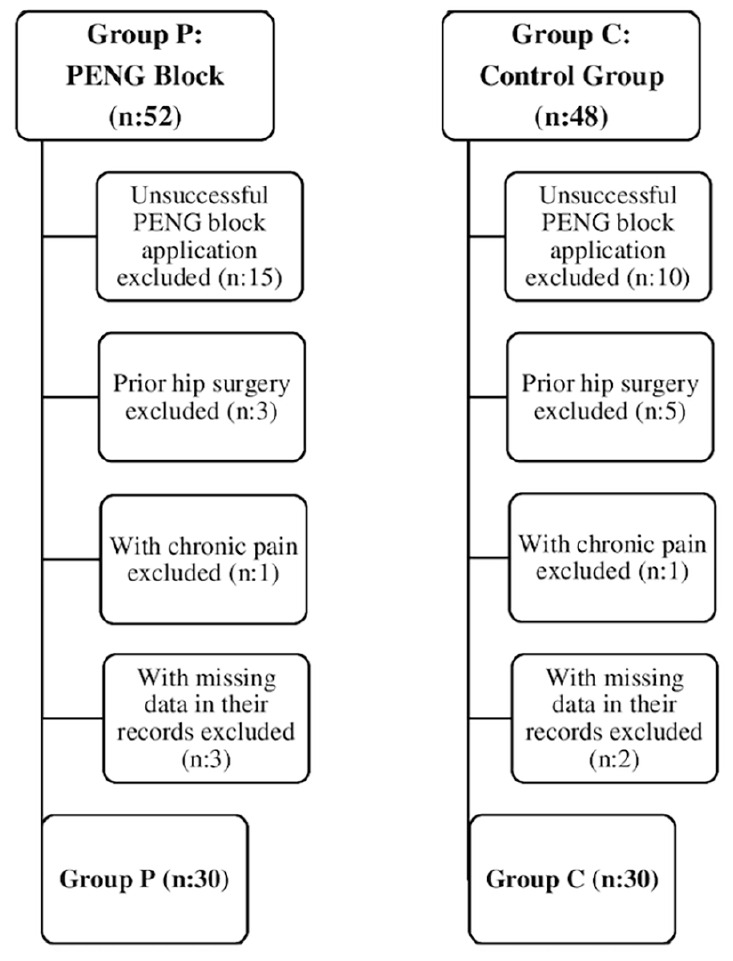
Patient flow chart of 100 patients undergoing hip surgery. PENG, pericapsular nerve group.

This retrospective study was performed after approval from the Clinical Research Ethics Committee of Süleyman Demirel University Faculty of Medicine (decision no.: 281570, dated: 10.06.2022). Between December 2021 and June 2022, 100 patients aged 18 and above in the American Society of Anesthesiologists’ (ASA) I-II-III risk group underwent hip fracture surgery using spinal anaesthesia at Süleyman Demirel University Faculty of Medicine Hospital. A statistical power analysis was conducted using data from similar studies as a reference. With an effect size of d=1.02 and an alpha error of 20%, the number of patients estimated to deliver the population with 80% power was calculated as 52. When patients who did not meet the study criteria were removed, a total of 60 patients, 30 with PENG block and 30 without block, were enrolled in the study. The data available from the files of all patients were included in the analysis. Patients with a history of chronic pain, previous hip joint surgery, failed PENG block application, and missing data in medical records were not included in the study (Figure 1).

Upon arrival at the preoperative care unit (PCU), patients were briefed about the procedure and written informed consent was obtained after an explanation. Standard ASA monitoring was then conducted following the acquisition of consent. Patients who demonstrated full cooperation were instructed about the numeric rating scale (NRS), and pain scores were registered on the pain follow-up form. In the absence of contraindications, all patients were administered a single dose of paracetamol (1 g). Following the assessment of the pain score in the PCU, a preemptive PENG block was performed or, alternatively, 10 mg tramadol was given intravenously as a single dose, followed by an infusion of 10 mg/hour, depending on the clinical condition of the patient. In group PENG (P), the linear USG probe for block was positioned in a direction parallel to the imaginary line crossing from the anterior inferior iliac spine and the iliopubic eminence. Using in-plane technique, an 80 mm peripheral block needle was inserted and 20 mL of 0.25% bupivacaine was injected to complete the block. In the control group (C), 10 mg intravenous bolus tramadol was given to patients who did not prefer block followed by 10 mg hr infusion in the PCU.

The NRS scores of all patients were registered and if the NRS score was >3, a 10 mg IV bolus of tramadol was given with a waiting period of 30 minutes before starting the surgical intervention. Following a 30-minute after the block, the patient was transferred to the operating room table.

In the absence of specific circumstances, patients underwent unilateral hypobaric spinal anaesthesia with a 25 G - 90 mm cutting-edge disposable spinal needle and received 1.5 mL of 0.5% bupivacaine (7.5 mg), 1.5 mL of distilled water, and 0.25 mL of fentanyl (12.5 µg). The number of attempts, success of dural puncture, and time of skin incision were noted on the anaesthesia follow-up form. Furthermore, the level of spinal anaesthesia was established by pinprick test at the 5^th^ minute.

Demographic data of the patients, surgical procedure and duration, the patient’s postoperative discharge site, heart rate, mean arterial pressure (MAP), and peripheral saturation (SpO_2_), values obtained before PENG block application, after PENG block application, during lateral decubitus positioning before spinal anaesthesia, after spinal anaesthesia and at postoperative discharge were recorded and evaluated. Similarly, NRS values before PENG block application, after PENG block application, during positioning before spinal anaesthesia, at the time of the patient was discharged from the operating room and at 2^nd^, 4^th^, 12^th^ and 24^th^ hours postoperatively, the duration of spinal anaesthesia (the time between the onset of spinal anaesthesia and skin incision), and the number of spinal anaesthesia attempts were noted and assessed. In addition, total perioperative tramadol consumption and postoperative complications (e.g., nausea and vomiting, hypotension, quadriceps muscle weakness, infection, haematoma, local anaesthetic toxicity), were noted and reviewed, from hospital information system data, anaesthesia tracking forms, operative notes, perioperative pain monitoring forms, and discharge notes.

### Statistical Analysis

This article presents the results of a statistical analysis of the data by using the Statistical Package for Social Science version 24. Qualitative data are presented as numerical values and percentages, while quantitative data are presented as means and standard deviations. The Kolmogorov-Smirnov and Shapiro-Wilk tests were used to determined the normality of the continuous variables. The Student’s t-test was used to evaluate the statistical significance of differences between two independent groups of normally distributed variables. The Mann-Whitney U test was employed to analyse the data obtained from two independent variable groups that did not have normal distribution. Finally, the chi-square test was applied to analyse categorical data.

## Results

The demographic and perioperative clinical characteristics and surgical durations of the cases were compared, in Table 1 (*P *> 0.05).

**Figure 2:**
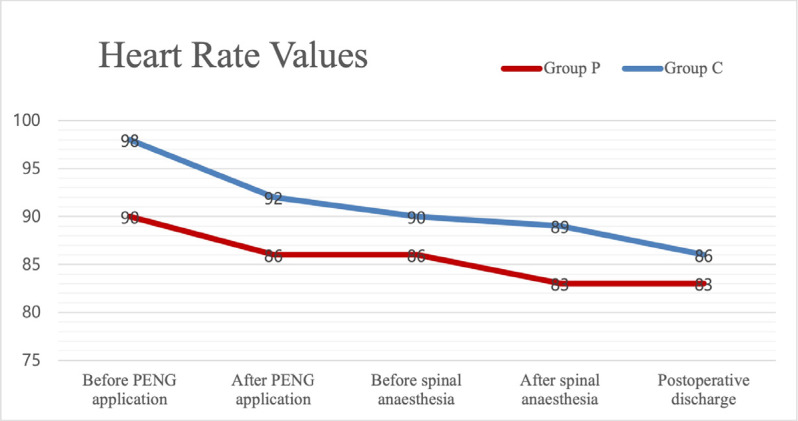
Heart rate values. Data are shown as mean. The Student’s t-test was used in the analysis of the independent variables PENG, pericapsular nerve group

**Figure 3:**
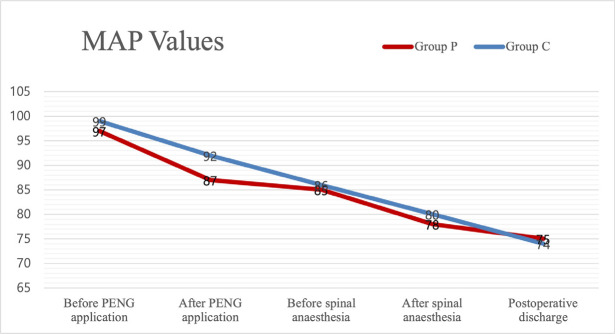
MAP values. Data are shown as mean. The Student’s t-test was used in the analysis of the independent variables MAP, mean arterial pressure; PENG, pericapsular nerve group

The number of patients requiring postoperative intensive care was five (8.3%), while the number of patients followed up in the ward was 55 (91.7%) (*P *> 0.05). Perioperative heart rate, MAP, and SpO_2 _values were not significantly different between the groups (*P *> 0.05) (Figures 2, 3).

**Figure 4:**
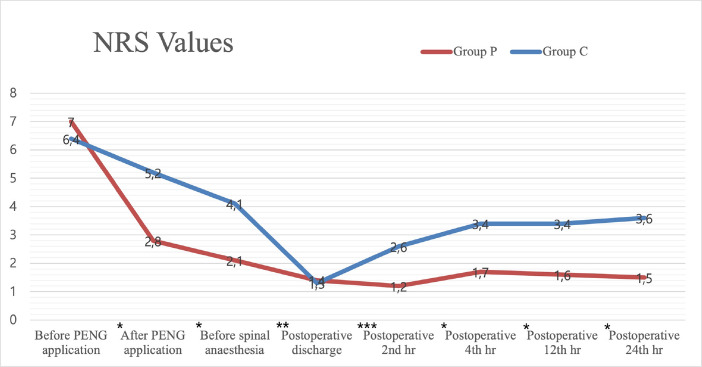
NRS values. Data are shown as mean. Mann-Whitney U test was used in the analysis of the variables NRS, numerical rating scale, ^*^P < 0.001, ^**^P=0.012, ^***^P=0.001

The NRS values before PENG block application were similar (*P *> 0.05). However, the NRS values after PENG block application, during positioning before spinal anaesthesia, and at the postoperative discharge, 2^nd^, 4^th^, 12^th^ and 24^th^ hours were found significantly lower in Group P than in Group C (Figure 4).

A statistically significant reduction in the duration of spinal anaesthesia and the number of attempts was observed in Group P (*P* < 0.001 and *P=*0.022, respectively) (Table 2).

A comparison of the number of spinal anaesthesia attempts between the groups revealed a statistically significant higher success rate on the first attempt in Group P, at 64% (*P*=0.023) (Table 3).

Nausea-vomiting and hypotension were observed only in Group C in a total of 6 patients and were statistically significant (*P* < 0.05). No instances of haematoma, bleeding, unintentional nerve injury, quadriceps muscle weakness, wound infection, local anaesthetic toxicity, or headache were observed in both groups.

A comparison of the tramadol consumption between the groups revealed that Group P exhibited a lower consumption rate (*P *< 0.001) (Table 4).

## Discussion

It has been shown that PENG block, which can be easily applied under USG-guidance before surgery in the supine position without requiring any change in the patient’s position, facilitates the application, shortens the duration and increases the success rate in the first attempt by providing a painless positioning to the patient during spinal anaesthesia application in hip fracture patients. Furthermore, this study showed that PENG block reduced postoperative pain, the need for opioids and the frequency of side effects.

Currently, there are no observational and comparative study investigating the duration and the number of spinal anaesthesia attempts with effective pain control using PENG block. In the literature, studies on PENG block are mostly in the form of case reports and case series.^9^ Our study is the first retrospective study to show that PENG block improves the success of spinal anaesthesia at the first attempt by reducing pain and shortening the duration of spinal anaesthesia.

A study conducted on more than 10 patients revealed that the average pain score, which was 7.5 before PENG block, decreased to an average of 1.2 when the patients were given spinal anaesthesia.^10^ The results of our study indicate that the average pain score, decreased from 7 before the PENG block to 2.8 after PENG block. Additionally, at the time of the lateral decubitus positioning before spinal anaesthesia, the average pain score was as low as 2.1. Consistent with the existing literature, NRS values ​​were significantly decreased after PENG block in Group P.

A randomised controlled study was conducted on 100 patients who underwent open prostatectomy. The effect of the spinal anaesthesia position on success was investigated. There was no significant difference in success between the two groups, with both demonstrating comparable outcomes. However, the number of attempts required was higher in the group that extended their legs to the table.^11^ We investigated the effect of providing pain control and increasing hip and knee flexion by giving the lateral decubitus position on the trial number and duration of spinal anaesthesia. Upon comparison of the data from both groups, the average NRS value was found to be 4.1 in Group C, with the average number of attempts was 2.2 and the average duration of spinal anaesthesia application was 18.1 minutes. In Group P, the average NRS value was 2.1, the average number of attempts was 1.6, and the average duration of spinal anaesthesia application was 13.1 minutes when the lateral decubitus position before spinal anaesthesia was employed. The hypothesis of effective pain control was achieved with a decrease in NRS values, during positioning and spinal anaesthesia application. Spinal anaesthesia success was increased at the first attempt, and application time got shorter in Group P.

In a prospective observational study involving 1647 patients, the initial puncture success rate was found to be 52.9%. The study included patients with an average age of 38 years and a majority of ASA I (1323) and a minority of ASA III (17). It was observed that male gender, difficulty in palpating spinous processes, presence of bone deformities, and lower experience level of the provider increased the number of attempts for a successful dural puncture.^12^ The initial puncture success rate of 45% observed in this study was lower than the 52.9% reported in previous studies. This discrepancy is believed to be due to the patient population, which presented greater challenges in administering spinal anaesthesia due to the structural changes of the spine associated with advanced age. The average age of the patients in this study was 74.7 years, and the ASA III patient ratio was 45%. Upon examination of the successful spinal anesthesia rate in the first attempt (Group P: 64%, Group C: 27%), it can be concluded that there is a significant difference between the two groups, confirming the hypothesis that PENG block increases the successful spinal anesthesia rate by facilitating the application of spinal anesthesia. Nevertheless, we also believe that further randomised controlled studies should be conducted in this regard.

Although femoral nerve block and fascia iliaca compartment block (FICB) have been demonstrated to have positive effects on perioperative analgesia, it is necessary to target the obturator nerve and accessory obturator nerve in order to achieve more effective pain control.^13^ It has been demonstrated that the blockade of the accessory obturator nerve and femoral nerve from the anterior capsule nerves plays a greater role than previously reported in providing pain control in hip fractures.^14, 15^ Girón-Arango et al.^8^ described a new regional anaesthetic technique, the PENG block, which was shown to result in a significant reduction in patients’ pain scores without quadriceps muscle weakness in five hip fracture patients. A randomised controlled study comparing FICB and PENG block in terms of motor function demonstrated that the PENG block was more effective in preserving motor function.^16^ Once more, the PENG block was demonstrated to be more efficacious than FICB in terms of postoperative analgesia.^17^

In our study, as in previous studies, pain control was achieved without quadriceps muscle weakness after PENG block.

It has been demonstrated that neuroaxial anaesthesia can reduce perioperative complication risks following total hip arthroplasty, regardless of age group and the presence of cardiopulmonary disease. Furthermore, the incidence of admission to the intensive care unit was lower in patients who received neuroaxial or neuroaxial plus general anaesthesia compared to those who received general anaesthesia in all groups.^18^ The rate of intensive care unit admission for patients undergoing hip fracture surgery in the literature is reported to be 32.5%.^19^ In our study, we found an intensive care unit admission rate of 8.3%, which we believe is due to the use of unilateral spinal anaesthesia to minimise haemodynamic changes.

The primary factor associated with increased mortality in general anaesthesia and spinal anaesthesia is intraoperative hypotension.^20^ A study of 90 patients found that the incidence of hypotension was lower in unilateral spinal anaesthesia (15%) than in bilateral spinal anaesthesia (56%).^21^ The administration of spinal anaesthesia in the lateral decubitus position with the fractured extremity positioned above the patient’s body due to the severe pain caused by movement in hip fracture patients has been found to prevent the exacerbation of pain on the fractured extremity and to enable the surgery to be performed without the patient needing to change position.

In an article comparing the haemodynamic effects of hypobaric spinal anesthesia in elderly patients over the age 80 of who underwent surgery for femoral neck fractures, it was shown that the use of moderate doses (6-7.5 mg) of bupivacaine provided advantages in terms of the onset and termination of motor block after surgery.^22^ Bupivacaine is the most extensively studied local anaesthetic for unilateral spinal anaesthesia, with minimal side effects.^3^ Consequently, the study opted for unilateral spinal anaesthesia with a moderate dose (7.5 mg) of bupivacaine. Neither group exhibited any significant alterations in haemodynamic parameters throughout the perioperative period. The combination of bupivacaine with 12.5 µg of fentanyl was selected in order to take advantage of the pain-reducing effect of fentanyl while limiting the use of systemic opioids.

The National Institute for Health and Care Excellence in the United Kingdom recommends that all patients with hip fractures receive pain management, irrespective of age or cognitive impairment. This should be initiated at admission and continued with paracetamol administered every six hours before and after surgery, with opioids added if pain persists. To prevent the administration of high doses of opioids, it is recommended that peripheral nerve blocks be employed.^23^ The results of our study indicated that postoperative NRS values were significantly lower and the total amount of tramadol consumed was significantly less in patients who received PENG block compared to the control group. These findings demonstrate that the PENG block provides effective analgesia and can be employed to reduce opioid consumption.

No serious adverse events, such as permanent nerve damage, haematoma, or local anaesthetic systemic toxicity, were observed in the patients who received a PENG block. In our study, six patients in Group C exhibited nausea, vomiting, and/or hypotension. We hypothesize that this is a side effect of postoperative tramadol use.

### Study Limitations

The study was subject to certain limitations, including its retrospective nature, the difficulty in accessing archive documents, the paucity of medical records, and the deficiencies in the history forms. Furthermore, the age of the patient population may have influenced the assessment of NRS values. Furthermore, postoperative analgesic consumption was not quantified using patient-controlled analgesia methods, and the end time of postoperative spinal anaesthesia was not monitored using the Bromage score. This may have resulted in challenges in pain assessment. There is a need for randomised controlled studies showing that preoperative PENG block application in hip fracture patients provides a more comfortable position to the patient during spinal anaesthesia, facilitates the application and increases the success of spinal anaesthesia.

## Conclusion

In our study, PENG block facilitated the administration of spinal anaesthesia by reducing pain, especially during patient transfer and spinal anaesthesia positioning, and also shortened the duration of spinal anaesthesia administration, thus increased the success of first attempt spinal anaesthesia. Moreover, PENG block reduced postoperative pain, opioid use, and side effects.

## Ethics

**Ethics Committee Approval:** This retrospective study was performed after approval from the Clinical Research Ethics Committee of Süleyman Demirel University Faculty of Medicine (decision no.: 281570, dated: 10.06.2022).

**Informed Consent:** Written informed consent was obtained.

## Figures and Tables

**Table 1. Demographic Data of Patients, Preoperative Clinical Characteristics and Distribution of Surgery Durations According to Groups:** 

**Variables**	**Group P (n = 30)**	**Group C (n = 30)**	**Total (n = 60)**	***P***
Age^*^	74.60±19.71	76.97±11.70	75.78±16.11	0.574
BMI^*^ (kg m^2-1^)	24.11±4.22	22.93±4.86	23.52±4.21	0.283
Gender^†^				
Male	15 (50.0%)	12 (40.0%)	27 (45.0%)	0.436
Female	15 (50.0%)	18 (60.0%)	33 (55.0%)	
ASA^†^				
1	6 (20.0%)	4 (13.3%)	10 (16.7%)	0.661
2	10 (33.3%)	13 (43.3%)	23 (38.3%)	
3	14 (46.7%)	13 (43.3%)	27 (45.0%)	
Surgical duration (min)	109.83±27.99	119.83±28.51	114.83±28.35	0.176

Data are shown as mean ± SD, number (%), ^*^t-test on independent variables, ^†^chi-square test, *P* < 0.05: Statistically significant

BMI, body mass index; ASA, American Society of Anesthesiologists Physical Condition Classification; min, minutes; SD, standard deviation.

**Table 2. Distribution of Spinal Anaesthesia Duration and Number of Attempts Between Groups:** 

**Spinal anaesthesia**	**Group P**	**Group C**	***P***
Duration (min)	13.63±2.49	18.13±3.08	<0.001
Attempts	1.63±0.96	2.27±1.11	0.022

Data are shown as mean ± SD, t-test was used in the analysis of the variables, *P *< 0.05: Statistically significant

SD, standard deviation; min, minutes.

**Table 3. Distribution of the Number of Spinal Anaesthesia Attempts Between Groups:** 

**Attempts**	**Group P (n = 30)**	**Group C (n = 30)**	**Total (n = 60)**	***P***
**1^†^**	19 (64%)	8 (27%)	27 (45%)	0.023^*^
**2^‡^**	5 (17%)	12 (40%)	17 (28%)	
**3**	4 (13%)	5 (17%)	9 (15%)	
**4**	2 (6%)	4 (13%)	6 (10%)	
**5**	0 (0%)	1 (3%)	1 (2%)	

Data are shown as number (%), chi-square test was used in the analysis of the variables,^ *^significant at 0.05 level according to exact chi-square test, ^†^post-hoc test is significant for attempts 1, ^‡^post-hoc test is significant for attempts 2, *P *< 0.05: Statistically significant.

**Table 4. Perioperative Tramadol Consumption Amount by Groups:** 

**Tramadol consumption (mg)**	**Group P (n = 30)**	**Group C (n = 30)**	***P***
**Total**	14.00±30.240	272.67±32.582	<0.001

Data are shown as mean ± SD, Mann-Whitney U test was used in the analysis of the variables, *P *< 0.05: Statistically significant

SD, standard deviation.
